# Tau Pathology and Adult Hippocampal Neurogenesis: What Tau Mouse Models Tell us?

**DOI:** 10.3389/fneur.2021.610330

**Published:** 2021-02-10

**Authors:** Sarah Houben, Mégane Homa, Zehra Yilmaz, Karelle Leroy, Jean-Pierre Brion, Kunie Ando

**Affiliations:** Laboratory of Histology, Neuroanatomy and Neuropathology, UNI (ULB Neuroscience Institute), Faculty of Medicine, Université Libre de Bruxelles, Brussels, Belgium

**Keywords:** neurogenesis, tauopathy, Alzheimer's disease, dentate gyrus, tau

## Abstract

Adult hippocampal neurogenesis (AHN) has been widely confirmed in mammalian brains. A growing body of evidence points to the fact that AHN sustains hippocampal-dependent functions such as learning and memory. Impaired AHN has been reported in *post-mortem* human brain hippocampus of Alzheimer's disease (AD) and is considered to contribute to defects in learning and memory. Neurofibrillary tangles (NFTs) and amyloid plaques are the two key neuropathological hallmarks of AD. NFTs are composed of abnormal tau proteins accumulating in many brain areas during the progression of the disease, including in the hippocampus. The physiological role of tau and impact of tau pathology on AHN is still poorly understood. Modifications in AHN have also been reported in some tau transgenic and tau-deleted mouse models. We present here a brief review of advances in the relationship between development of tau pathology and AHN in AD and what insights have been gained from studies in tau mouse models.

## Introduction

**Figure 1:**
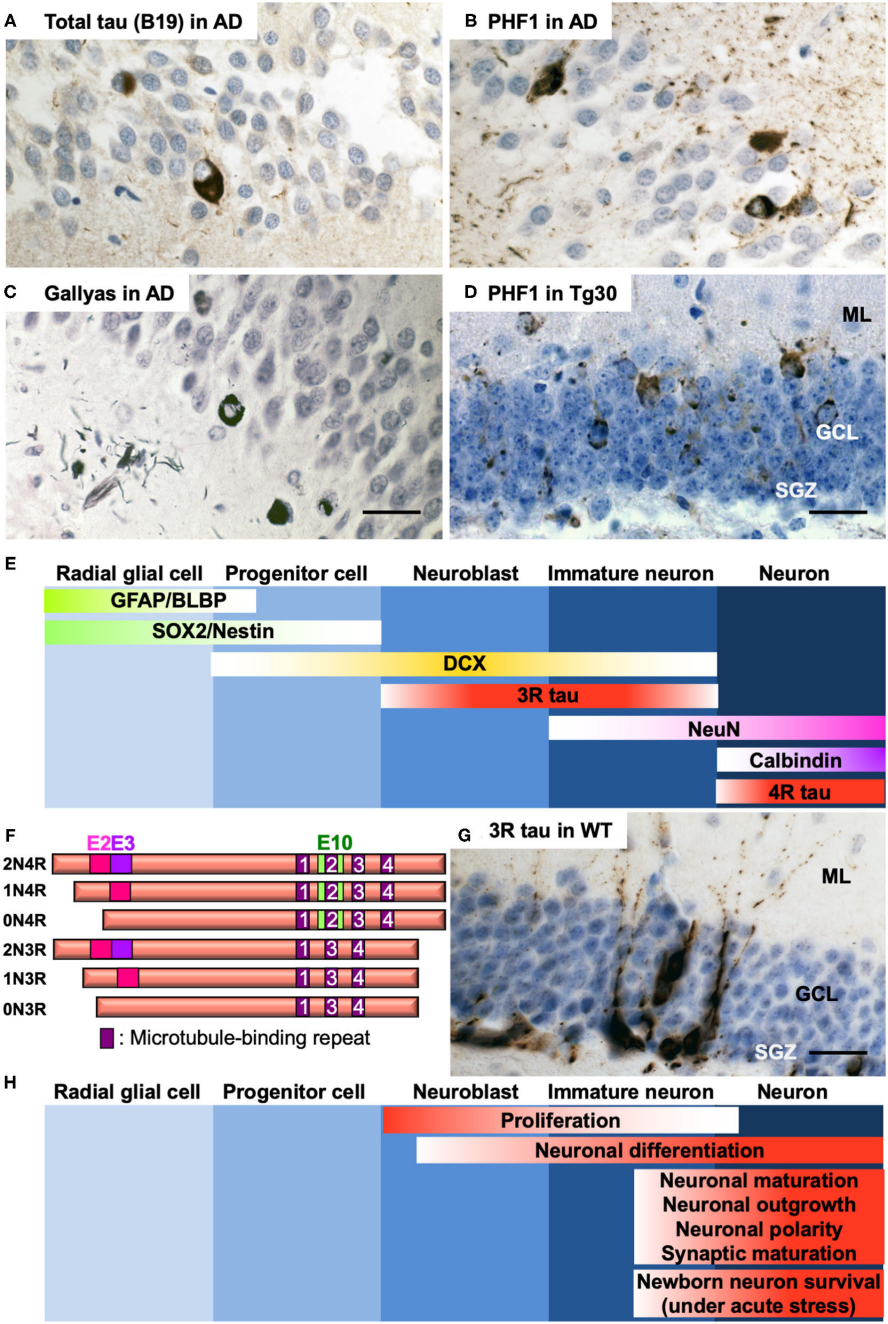
**(A–C)** Representative photos of tau pathology detected in the DG of *post-mortem* brain section of a 65-year-old male AD patient (Braak VI). Tau pathology was detected by anti-total tau B19 antibody (9) **(A)**, anti-phospho Ser396/404 tau PHF1 antibody (10) **(B)**, or by Gallyas silver staining (11) **(C)**. **(D)** Representative photo of tau pathology detected by PHF1 in the DG of 12-month-old tau Tg30 mice (12). ML, molecular layer; GCL, granule cell layer; SGZ, subgranular zone. **(E)** Specific markers for five different stages of AHN in the dentate gyrus of the hippocampus. GFAP, glial fibrillary acidic protein; BLBP, brain lipid-binding protein; SOX2, SRY (sex determining region Y)-box 2; DCX, doublecortin; 3R tau, tau with 3 repeats of microtubule-binding sequences; NeuN, neuronal nuclei; 4R tau, tau with 4 repeats of microtubule-binding sequences. **(F)** Schematic representation of the human 6 isoforms of tau protein. Exon 2, 3, and 10 (E2, E3, and E10, respectively) are alternatively spliced. Alternative splicing leads to 0, 1 or 2 inserts near amino terminus (0N, 1N, or 2N, respectively) and 3 or 4 repeats (3R or 4R, respectively) of microtubule-binding sequences near carboxyl terminus. The shortest 0N3R isoform is predominantly detected in immature neurons of fetal brains and of adult hippocampus. While only 4R isoforms are principally expressed in adult mouse brains, all the 6 isoforms are expressed in adult human brains. **(G)** Immunostaining of immature neurons by anti-3R tau RD3 antibody (Merck Millipore #05-803) in the dentate gyrus of the hippocampus in a 12-month-old wild-type mouse. **(H)** Functional involvement of tau at different stages of AHN. Hematoxylin counterstaining for **(A–D,G)**. Detailed protocol on histological analyses is available in (12). Scale bars: 25 μm.

AD has two neuropathological hallmarks, amyloid plaques, and NFTs. Amyloid plaques are composed of amyloid ß peptides (1) derived from successive cleavages of amyloid precursor protein (APP) (2). NFTs are constituted of microtubule-associated protein tau (MAPT) (3). In a family of neurodegenerative diseases called tauopathies including AD, tau undergoes hyperphosphorylation and aggregation to develop pathological forms of tau species such as oligomers or highly insoluble filaments that form NFTs. The levels of NFTs are highly correlated with cognitive decline (4). Tauopathies include frontotemporal lobar degeneration (FTLD) with tau positive inclusions with or without gene mutation in *MAPT*, Pick disease, progressive supranuclear palsy, corticobasal degeneration, and others (5). In AD brains, tau deposition occurs in a stereotypical manner, with the hippocampus, limbic structures, brain stem, and the basal nucleus of Meynert being most affected at the early stages (6). The hippocampus is a crucial brain structure for the acquisition of new memories and retrieval of older memories. Afferent pathways to the dentate gyrus (DG) are affected by NFTs developing in the entorhinal cortex (6), and NFTs develop in the granule cell layer (GCL) (7, 8) in the DG in AD and in some tau transgenic mouse models (Figures 1A–D). Tau pathology in the DG might play a role in memory impairment. Whereas, abnormalities in AHN have been extensively investigated in AD mouse models based on *APP* or *PSEN1/2* familial AD mutations (13, 14), the impact of tau pathology on AHN remains largely unclear in AD and other tauopathies. We provide here a brief overview of recent advances on the relationship between development of tau pathology and AHN in AD and what insights have been gained from studies in tau transgenic mouse models.

## Normal AHN

Since its discovery in mammalian brain in 1965 (15), AHN has been documented in many species (16–20). In placental mammals and marsupials, adult neurogenesis is mainly limited to two areas: the subventricular zone (SVZ) along the lateral ventricles and the subgranular zone (SGZ) of the dentate gyrus (DG). AHN is necessary for spatial memory and specific learning tasks and is related to mood regulation (21, 22).

Neural stem cells found in the SGZ of the hippocampus generate new neurons for the DG (23). The identity of adult neural stem cells remains still controversial. Growing evidence suggests that they have an astrocytic phenotype (24, 25) or they may be radial glial cells, able to give rise asymmetrically either to a glial cell or a neuron (26). There are five principal developmental stages of AHN starting from the radial glia-like cells, progenitor cells, neuroblast cells, immature neurons, and finally mature neurons as granular cells (27). These stages can be identified by specific markers such as GFAP, BLBP, SOX2, Nestin, Doublecortin (DCX), tau with three-repeats (3R) or four-repeats (4R) of microtubule-binding repeat domains (RD), NeuN, and Calbindin (Figure 1E) (23, 28). Newborn cells can be experimentally traced using exogenous cell tracers such as thymidine analogs that are incorporated into dividing cells during DNA synthesis (29). Newborn neurons can be also identified by other mitotic markers such as Ki67 in combination with neuronal markers (30). Studies have provided compelling evidence for the persistence of AHN in humans and non-human primates (31, 32). There are some contradictory findings pointing to hardly detectable levels of AHN in human brains due to a sharp decrease in childhood (33, 34). A breakthrough was made when a study provided evidence for the birth of ~700 newborn neurons a day per one adult human hippocampus by measuring the concentration of nuclear bomb test-derived ^14^C in genome DNA (35). By a similar approach, striatum has also been recently identified as a neurogenic zone in the adult human brain (36). Annual turnover rates are estimated as 1.75% of neurons in the hippocampus and 2.7% in the striatum in the human adult brain (35, 36). Although observed in other species than human (37, 38), the role of adult striatal neurogenesis remains largely elusive (39).

Stress, aging, and disease have a negative impact on AHN (40). On the contrary, AHN can be enhanced in rodents by lifestyle factors such as environmental enrichment (EE) (41), physical activity (e.g., running) (42, 43), anti-depressants (44), or electroconvulsive seizures (45).

## TAU Proteins. “Canonical” and “Non-canonical” Functions

Tau is a cytosolic protein predominantly expressed in neurons. Tau has physiological roles, the most studied being the regulation of the axonal transport and of the cytoskeleton by maintaining the stability of microtubules (46). Human *MAPT* gene is located on chromosome 17 and contains 16 exons. Exons 2, 3, and 10 are alternatively spliced to give rise to six different isoforms in the adult human central nervous system (Figure 1F) (47). Alternative splicing of exon 10 results in generating either tau with 3R or 4R microtubule-binding sequences in the half carboxyl domain. 3R and 4R tau isoforms include sequences of exon 2, exons 2 and 3, or none of them in their amino domain. Tau regulates axonal microtubule assembly but has also other functions (48) by interacting with many partners in addition to microtubules (49, 50). Among other functions, tau is implicated in pathways regulating synaptic plasticity, cell signaling, and DNA integrity (51). Tau is also secreted via several pathways (52), a process that is thought to play a role in the “Prion-like” propagation of tau pathology (53) but that is not well-understood in physiological conditions. This multifunctional aspect of tau might be involved in the regulation of AHN.

## Developmental Evolution of Tau Protein Expression and Role of Tau in AHN

While six isoforms are expressed in adult human brain, only 4R isoforms are predominantly detected in the mature neurons of mouse brains. During brain development, only the 0N3R isoform (fetal isoform) is expressed in human and rodent brains (54, 55). Owing to the lack of one microtubule-binding domain, 3R tau isoforms have less affinity for microtubules and consequently less efficiency to promote microtubule assembly compared to 4R isoforms (56). Expression of 3R tau isoforms is thus related to plasticity in neuronal development in neonatal stage and in neurogenesis for dynamic process formation, neurite elongation, and neuronal polarity (57–59). 3R tau isoform lacking exon 2 and 3 is also expressed in the adult brain in the immature neurons in the SGZ (60) and can be used as a specific marker to detect newborn neurons and newly generated axons in the adult mouse hippocampus (28, 61). The number of cells expressing 3R tau isoforms in the SGZ decreases with age in mice, but they are still detectable at 12 months (Figure 1G) (12). Tau in immature neurons in the SGZ shows a higher phosphorylation seemingly through activated GSK-3 (62), reducing its affinity for microtubules and providing these cells with a more dynamic microtubule network during dendritic and axonal outgrowth. In these immature neurons, tau is abundant in the somatodendritic domain (as during development) and appears to be at least partly in a microtubule-unbound form (63). Increased tau phosphorylation is associated with increased proliferation of newborn neurons (62).

## AHN in AD

Emerging evidence suggests that overall AHN (e.g., generation of fully functional new neurons) is reduced in AD (64). The detection of AHN markers by immunohistochemistry on *post-mortem* brain tissues has recently confirmed the existence of AHN in aged healthy subjects and a significant reduction of DCX-positive immature neurons in AD brains (65, 66). AHN drops sharply even at the early stage of cognitive decline in the patients with mild cognitive impairment (66). These studies imply that the reduction of AHN may directly compromise cognitive functions (67). Importantly, SOX2-positive neural stem cells were increased in some cognitively normal subjects but with extensive AD neuropathological lesions (68), implying that increased AHN may rescue cognitive deficits caused by AD lesions. Numerous genetic factors and variants implicated in AD (*Apolipoprotein E, PSEN1, APP*) have been identified with a modulating role on AHN in human AD patients (69). This observation is supported by the generalized decrease in newborn neuron generation observed in various AD transgenic mouse models overexpressing FAD-related mutant *APP* and/or *PS1* (13) or overexpressing APP intracellular C-terminal domain fragments (AICD) (70).

## AHN in TAU Mouse Models

Studies of AHN in different tau transgenic mouse models have suggested that tau has critical roles in proliferation, neuronal differentiation/maturation, dendritic/axonal outgrowth, neuronal plasticity and synaptic maturation in DG. Tau is also involved in selective cell death of newborn granule neurons in case of acute stress (71) (Figure 1H). However there remain controversies in distinct tau models (Table 1). Whereas tau knockout mice are viable and macroscopically normal (72, 73, 92), behavioral studies have unraveled that they exhibit abnormalities such as hyperactivity (93) and deficits in short-time memory in an age-dependent manner (94). Deletion of endogenous tau also leads to delayed neuronal maturation in primary cultured neurons (73) and transcriptional repression of neuronal genes in the hippocampus (95). A significant reduction of DCX- and NeuroD- positive neuroblast cells in tau knockout mice was observed (62). On the contrary, Criado-Marrero et al. have recently reported that BrdU-positive newborn cells and DCX-positive immature neurons were increased in the DG and SVZ of tau knockout mice at 14 months (75). Yet, other two independent studies have reported that DCX-labeled neuroblast cell number was not altered in the DG of adult tau knockout mice (71, 74). Moreover, tau has critical roles in both stress-induced suppression of AHN and stimulatory effect of EE. Unlike wild-type mice, tau knockout mice are insensitive to the modulation of AHN by stress or EE (71).

**Table 1:** Summary of neurogenesis changes in tau mouse models.

**Mouse line**	**Tau expression**	**Neurogenesis assessment**	**References**
**Tau knockout models**			
tau^−/−^ Tucker et al. (72)	–	Decrease in the number of DCX- and NeuroD- positive cells (age not indicated)	(62)
tau^−/−^ Dawson et al. (73)	–	No change in the number of BLBP, Sox2- and DCX positive cells at basal conditions but reduction of dendritic and synaptic maturation of newborn granule neuron (4 months)	(71)
tau^−/−^ Dawson et al. (73)	–	No change in the number of DCX/BrdU double positive cells (9 weeks)	(74)
tau^−/−^ Dawson et al. (73)	–	Increase in the number of DCX- and BrdU- positive cells (14 months)	(75)
**Human non-mutant tau models**			
KOKI Terwel et al. (76)	2N4R human non-mutant tau in the absence of murine tau	Increase in the number of DCX- and BrdU-positive cells (2 months)	(77)
hTau Andorfer et al. (78)	6 isoforms of human non-mutant tau in the absence of murine tau	Decrease in the number of DCX-, Ki67-, and BrdU-positive cells (2, 6, 12 months)	(79)
Injection of human tau-Cy5 in WT mice	Endogenous murine tau and injected monomeric 2N4R human non-mutant tau	No change in the number of DCX-positive cells but change in the morphology of newborn granule cells	(80)
AAV-mediated expression of human tau	Human tau overexpressed in DG interneurons	Decrease in the number of BrdU-positive cells and DCX-positive cells	(81)
Lentiviral expression of human tau in hilar astrocytes	1N3R human non-mutant tau overexpressed in hilar astrocytes in the presence of murine tau	Decrease in the number of DCX-positive cells	(82)
**Human FTLD-mutant tau models**			
THY-Tau22 Schindowski et al. (83)	IN4R human double mutant G272V/P301S tau in the presence of murine tau	Increase in the number of DCX- and BrdU-positive cells (6 months)	(84)
Tg30 Leroy et al. (85)	IN4R human double mutant G272V/P301S tau in the presence of murine tau	Decrease in the number of DCX-, Ki67-, and tau 3R-positive cells (12 months)	(12)
Tg30/tauKO Ando et al. (86, 87)	IN4R human double mutant G272V/P301S tau in the absence of murine tau	Increase in the number of DCX-positive cells (12 months) compared to Tg30 and wild-type mice	(12)
Tau^VLW^ Lim et al. (88)	2N4R human triple mutant G272V/P301L/R406W tau in the presence of murine tau	Decrease in the number of DCX- and IdU- positive cells (2 months)	(89)
**Tau repeat-domain models**			
Tau^RDDK^ and tau^RDDKPP^ (90)	Tau^RDDK^ expressing pro-aggregant mutant tau repeat domain and tau^RDDKPP^ expressing anti-aggregant mutant tau repeat domain	Decrease in hippocampal volume at 16 months in tau^RDDK^. Increase in hippocampal volume at 16 months, in hippocampal stem cell proliferation and in the number of DCX-positive cells in tau^RDDKPP^	(91)

Human non-mutant tau seems to have several roles in AHN such as suppressing proliferation and promoting neuronal differentiation. KOKI mice expressing human 2N4R tau isoform in the absence of murine tau (76) had an increase in DCX-positive immature neurons, hippocampal volume and cell number in DG and an improved cognitive function (77). Nevertheless, other studies suggest negative effect of human non-mutant tau on AHN in mouse brains. hTau mice expressing the 6 isoforms of non-mutant wild-type human tau (78) in the absence of murine tau had reduced DCX-positive immature neurons at 2 and 6 months (79). Hippocampal injection of soluble non-mutant 2N4R human tau led to morphological changes of newborn granule neurons without changing the total number of DCX-positive neuroblast cells (80). Adeno-associated virus-mediated specific overexpression of human tau in DG interneurons induced deficits in AHN by suppressing GABAergic transmission (81). Another recent study has reported an impact of glial tau accumulation on AHN. Lentiviral-mediated 1N3R tau accumulation in hilar astrocytes in mouse led to reduction of AHN accompanied by impaired spatial memory performances (82).

Abnormalities in AHN have been observed in FTLD-mutant tau transgenic mouse models. In THY-Tau22 and Tg30 mice that express a human 1N4R tau mutated at G272V and P301S under a Thy1.2-promoter, Gallyas-positive NFTs are detectable from 6 months in hippocampus (83, 85). An increase in AHN was observed with the DCX and BrdU markers in 6-month-old THY-Tau22 mouse (84). Nonetheless, Tg30 mice exhibited an impaired AHN at 12 months, an age in which some of the granule cells in DG have a severe somatodendritic tau pathology (Figure 1D) (12). By crossing Tg30 with tau knockout mice (72), we generated Tg30/tauKO mice that express only human mutant tau in the absence of murine tau (86, 87). The reduction of AHN observed in Tg30 mice at 12 months was rescued in the Tg30/tauKO mouse model as measured by DCX-positive cell number (12). Another independent study reported that Tau^VLW^ mice carrying G272V, P301L, and R406W mutant tau (88) also had decreased DCX-positive immature neurons (89). Interestingly, EE significantly increased the number of DCX-positive immature neurons in wild-type littermates but not in Tau^VLW^ mice (89). To our knowledge, this is the first and only report showing that tau pathology may inhibit the response to a positive factor enhancing AHN.

The overall controversies may derive from the variation in the age and from the heterogeneities of tau species in distinct models. Tau^RDDKPP^ mice expressing anti-aggregant tau RD showed increased number of DCX-positive cells in DG and a larger volume of hippocampus unlike tau^RDDK^ mice expressing pro-aggregant mutant tau RD (91). The latter findings support the idea that distinct tau species seem to have different effects on neurogenesis.

## Discussion

There are conflicting reports as to whether AHN persists in late age in humans. Controversies may be partially due to the limited availability of adequately preserved *post-mortem* human brain samples. The technical and methodological issues can further add variability in detecting specific markers of neural stem and progenitor cells in human autopsy tissues. Some of the conflicting results are also presumably related to the heterogeneities in individual life stories: age, sex, lifestyle, physical activities, with or without previous disease histories, and medical status at the end of life. There is a great variability in the *post-mortem* delays and processing methods of human *post-mortem* brain tissues. In general, fixation is known to play a critical role in antigen preservation since some epitopes are more prone to denaturation during the fixation. For example, the immature neuron marker DCX undergoes rapid degradation during the *post-mortem* period (96). Some difficulties could be overcome by tightly documenting the brain samples and their processing, optimizing the methodologies (65), and standardization of detailed protocols (97).

Although tau seems involved in modulating AHN, there are controversies among the different tau mouse models about the effect of tau ablation or overexpression. As for human samples, controversial reports may derive from distinct protocols and various parameters such as genetic background, age, gender, and tau species. Distinct time point of analysis could lead to data variation (98). A remarkable sex difference was observed in AHN of rodent brains (75, 99). Furthermore, data variability may be caused by the sensitivities of antibodies used for detection (29). Besides, the methods of analysis and quantification have significant impact on the results. One of the most commonly used approaches is to measure total proliferating cell number using optical fractionator, an unbiased stereological method, on serial sections of the whole hippocampus (100). Since the distributions of the proliferating cells are not homogeneous and are often in the form of clusters in the SGZ of hippocampus (30), measuring setup needs to be carefully optimized (101).

The mechanisms behind AHN impairment in AD are still poorly understood. Numbers of independent studies have shown that amyloid pathology, APP, and PSEN1/2 are involved in modulating AHN in AD transgenic mouse brains (13). Since tau pathology led to defects in AHN in several tauopathy mouse models (12, 89, 91), we support the idea that tau pathology impairs AHN independently from amyloid pathology. In this context, it would be highly informative to study AHN in the *post-mortem* human brains of primary tauopathies devoid of amyloid pathology (e.g., FTLD with tau pathology, etc.). Yet, more studies are necessary to better understand both physiological and pathological roles of tau in AHN.

Given that increased AHN is associated with preservation of cognitive functions in non-demented individuals with AD lesions (68), stimulation of AHN should be beneficial. However, tau pathology presumably plays a negative role in AHN: EE led to increased AHN in wild-type mice but not in tau^VLW^ transgenic mice (89). Taking into consideration that an ablation of murine tau rescued AHN impairment in Tg30 mice (12) and stress-induced suppression of neurogenesis (74), reduction of tau may be beneficial for AHN. Indeed, there is compelling evidence showing the efficacy of tau reduction via anti-sense oligonucleotides (ASOs) to prolong life expectancy, reduce tau pathology, and rescue behavioral deficits in tau transgenic mice (102). Cautions need to be taken as complete ablation of tau leads to deficits in cognitive function in an age-dependent manner (94). Tau is a multifunctional protein and the net benefit of long-term reduction of tau still remains unclear (48). There are numbers of factors that can boost AHN such as EE, physical activities, or pharmacological agents (44). Testing these in tau transgenic models of tauopathies in combination with modulation of tau expression may open a new window for future therapies.

## Author Contributions

SH, J-PB, and KA wrote the main manuscript. All the authors participated in constructing the concept and writing the manuscript, contributed to manuscript revision, and read and approved the submitted version.

## Conflict of Interest

The authors declare that the research was conducted in the absence of any commercial or financial relationships that could be construed as a potential conflict of interest.
